# Proposal of neural network model for neurocognitive rehabilitation and its comparison with fuzzy expert system model

**DOI:** 10.1186/s12911-023-02321-1

**Published:** 2023-10-16

**Authors:** Martin Kotyrba, Hashim Habiballa, Eva Volna, Robert Jarusek, Pavel Smolka, Martin Prasek, Marek Malina, Vladena Jaremova

**Affiliations:** 1 Department of Informatics and Computers , University of Ostrava, Faculty of Science, 30.dubna 22, Ostrava, 70103 Czech Republic; 2 University Hospital of Ostrava, 17. listopadu 1790/5, Ostrava, 70852 Czech Republic

**Keywords:** Neural network model, Fuzzy expert system model, Neurocognitive rehabilitation, ACE-R, CHC

## Abstract

This article focuses on the development of algorithms for a smart neurorehabilitation system, whose core is made up of artificial neural networks. The authors of the article have proposed a completely unique transfer of ACE-R results to the CHC model. This unique approach allows for the saturation of the CHC model domains according to modified ACE-R factor analysis. The outputs of the proposed algorithm thus enable the automatic creation of a personalized and optimized neurorehabilitation plan for individual patients to train their cognitive functions. A set of tasks in 6 levels of difficulty (level 1 to level 6) was designed for each of the nine CHC model domains. For each patient, the results of the ACE-R screening helped deter-mine the specific CHC domains to be rehabilitated, as well as the initial gaming level for rehabilitation in each domain. The proposed artificial neural network algorithm was adapted to real data from 703 patients. Experimental outputs were compared to the outputs of the initially designed fuzzy expert system, which was trained on the same real data, and all outputs from both systems were statistically evaluated against expert conclusions that were available. It is evident from the conducted experimental study that the smart neurorehabilitation system using artificial neural networks achieved significantly better results than the neurorehabilitation system whose core is a fuzzy expert system. Both algorithms are implemented into a comprehensive neurorehabilitation portal (Eddie), which was supported by a research project from the Technology Agency of the Czech Republic.

## Introduction, motivation and research focus

Neurocognitive rehabilitation (NR) can be defined as a methodical approach to correcting cognitive deficiencies, based on a thorough assessment and understanding of the cognitive impairments caused by brain damage. The concept of NR, in its broader sense, aims to assist individuals in coping with mental deficits, regaining self-control, returning to work, and enjoying leisure activities, among other things. NR should be an integral part of the comprehensive rehabilitation process for individuals with brain damage, and should involve a multidisciplinary approach. We define it as a set of diagnostics, therapeutic, preventive, and organizational measures aimed at maximizing an individual’s functional capacity and creating optimal conditions for their integration into normal social and economic life [1]. Therefore, neurocognitive rehabilitation draws from knowledge in the fields of neuropsychology, cognitive psychology, behavioral psychology, as well as occupational therapy, language and speech therapy, and special education and can be considered one of the most dynamic areas with continuously expanding theoretical, methodological, and application spheres.

However, in the early stages of treatment, the use of relatively demanding neuropsychological diagnostic methods may not be possible for patients. So how can rehabilitation be carried out in the absence of diagnostic results? The answer can be found in this article.

Authors of the article developed a neurorehabilitation system incorporates artificial intelligence that can take the results of a basic ACE-R screening test administered and transfer them to the CHC model domains. The proposed neurorehabilitation system automatically generates a cognitive rehabilitation plan based on the CHC model, following a screening test (ACE-R) administered at the bedside.

The transformation of ACE-R results into CHC model domains was achieved by modifying the factor analysis according to Connolly et al. [2]. Authors of the article published this topic in [3]. Expert systems were used there as the artificial intelligence model, which were adapted and verified on real patient data.

However, authors of the study also have extensive experience with applications of artificial neural networks. Therefore, this article describes the proposed a personal neurorehabilitation system based on artificial neural networks that were used there as the artificial intelligence model. We used the same real dataset as in [3] for our experimental validation study and we compared obtained results with outputs from the expert system.

### Neurorehabilitation using ICT techniques and cases

Neurorehabilitation using ICT tools involves using information and communication technologies to support the rehabilitation process of patients with neurological disorders. This method is becoming increasingly popular due to its effectiveness and benefits for patients. ICT tools can be used for various types of neurorehabilitation, including physiotherapy, speech therapy, occupational therapy, and cognitive therapy. Among the most commonly used ICT tools are *virtual reality* [4], *games* [5], *smartphone and tablet applications* [6], *sensors, and robots* [7].

Cognitive function training is used to improve various areas of cognitive functioning, such as attention, memory, information processing speed, problem solving, and more. This type of training can be useful for people with neurological conditions such as Alzheimer’s disease, Parkinson’s disease, multiple sclerosis, and many others. There are many different ways to train cognitive functions [8], including computer programs, mobile phone and tablet applications, paper exercises, and more. These tools can be used in various settings, including the home environment, schools, or healthcare facilities. Cognitive function training can be individually tailored to each patient and can be performed in combination with other types of therapy, such as physical therapy, occupational therapy, or speech therapy. The advantage of cognitive function training is that it can be performed at different levels of difficulty and intensity, so it can be adapted to the individual needs of each patient.

Neurorehabilitation using ICT tools has several advantages. These tools provide patients with the opportunity to train their abilities in a safe and controlled environment. Furthermore, they allow the monitoring of the patient’s progress in real-time and provide therapists and doctors with more information about the patient’s condition and needs. Patients may feel more motivated and more involved in the rehabilitation process due to the interactive nature of these tools.

Research has shown that cognitive function training can be an effective way to improve cognitive functions in people with neurological disorders. However, it is important for the training to be tailored to the individual needs of the patient and performed under the supervision of a qualified professional. The following is a categorization of publications according to the most commonly used ICT tools in neurorehabilitation.

#### Virtual reality

Virtual reality (VR) interventions are increasingly used in individuals with brain injuries. This publication [9] focuses on the efficacy of cognitive rehabilitation in patients with Parkinson’s disease. It provides a critical overview of cognitive rehabilitation research with an emphasis on the effects on patients’ cognition and brain. The authors focus on different approaches to rehabilitation, such as memory training, attention, and executive functions. They also address various rehabilitation methods, including traditional methods and new technologies such as virtual reality. The authors point out that cognitive rehabilitation can be a useful tool for improving cognitive functions in patients with this disease, but the effectiveness of rehabilitation depends on several factors, such as the degree of disease progression and individual patient characteristics. Work [10] details leading-edge applications of virtual reality across a broad spectrum of psychological and neurocognitive conditions. Tracks the contributions of VR devices, systems, and methods to accurate assessment, evidence-based and client-centered treatment methods. The objective of this study [11] was to determine the effects of VR on overall cognitive functioning in individuals with neurocognitive disorders. Authors conducted a systematic review of the published literature on immersive and nonimmersive VR technologies targeting cognition in minor and major neurocognitive disorders. A total of 564 individuals with neurocognitive disorders were included in the review.

#### *Apps for smartphones and tablets*

The publication [12] provides important information on the use of computer-based cognitive function training in advanced age and demonstrates the potential of this method for improving the quality of life of older people. The publication [13] deals with the use of brain-computer interfaces (BCI) in neurorehabilitation after a stroke. BCI is a technology that allows for direct communication between the brain and the computer using EEG. The authors focus on the possibilities of using BCI in neurorehabilitation of patients after a stroke. They discuss various applications of BCI, such as training cognitive functions, motor rehabilitation, communication, and improving the quality of life. They also address the benefits and challenges of using BCI in neurorehabilitation, such as technical problems, uncertain clinical outcomes, and ethical issues. This article [14] discusses some of the many opportunities and challenges of implementing configured built-in sensors on mobile devices to enhance assessments and monitoring of neurocognitive functions as well as disease progression across neurodegenerative and acquired neurological conditions. Built-in sensor information on mobile devices is found to provide information that can enhance neurocognitive assessment and monitoring across all functional categories. Configurations of positional sensors (e.g. accelerometer, gyroscope, and GPS), media sensors (e.g. microphone and camera), inherent sensors (e.g., device timer), and participatory user-device interactions (e.g. screen interactions, metadata input, app usage, and device lock and unlock) are all helpful for assessing these functions for the purposes of training, monitoring, diagnosis, or rehabilitation.

#### *Senzors and robots*

The publication [15] focuses on the perspectives of using robotics in neurorehabilitation and shows that this field has the potential to bring significant innovation and improve rehabilitation outcomes in patients with neurological disorders. The authors focus on the use of robotics in neurorehabilitation and discuss how robots could contribute to improving rehabilitation effectiveness. They pay particular attention to the development of new robotic technologies that allow for personalized and effective rehabilitation. This systematic review [16] presents the state of the art of wearables used by Parkinson’s disease patients or the patients who are going through a neurocognitive disorder. The research findings proved that sensor based wearable devices, and specially instrumented insoles, help not only in monitoring and diagnosis but also in tracking numerous exercises and their positive impact towards the improvement of quality of life among these patients.

#### *Games and SW tools*

The publication [17] focuses on improving cognitive rehabilitation after traumatic brain injury through personalized telerehabilitation services. The authors developed the Guttmann personal trainer software tool, which consists of two main parts: software applications and a special device for monitoring and providing feedback on patient performance. The software application contains a range of games and exercises designed to strengthen cognitive functions such as attention, memory, reaction times, and information processing speed. Exercises are personalized based on the specific needs and cognitive abilities of the patient. The monitoring and feedback device consist of a touch tablet and special sensors that measure reaction times and other performance parameters of the patient. These data are used to provide feedback to the patient and their healthcare provider to adjust the rehabilitation program accordingly. The publication [18] deals with the use of mobile games for assessing the level of cognitive impairment in patients. The authors present that game performance in mobile games can provide a sufficiently reliable and accurate method for assessing the level of cognitive impairment. The paper presents the results of an experiment in which a new method for assessing cognitive functions in patients with neurological diseases was tested. Special mobile games were used, which were designed for easy and enjoyable interaction with patients and at the same time served to obtain data for evaluating their cognitive functions. The proposed game called “Match: Remembering Cards” tests the memory and attention of the patient and also allows for remote diagnosis of the patient’s condition. The proposed game called “Find the Route” tests the spatial thinking and navigation abilities of the patient. This game uses a virtual environment, and the patient must find the way between different points in space. The proposed game called “Catch the Apples” tests reaction speed and movement coordination. The game includes different levels of difficulty to be used for different levels of cognitive performance of the patient. All of these games were designed to provide a fun and interactive way to evaluate the cognitive functions of patients, especially those with Alzheimer’s disease, and to enable remote diagnosis and monitoring of their condition. In the publication [19], the authors deal with the design and development of an online cognitive training system that aims to provide training in cognitive abilities such as memory, attention, and reaction speed. The system consists of a series of games that are adapted to the cognitive performance level of individual users. The games are designed to be fun, motivating, and challenging to stimulate the brain and improve cognitive function. Each game has a certain level of difficulty that gradually increases as the user improves their skills. The system also includes an adaptive training function, which allows the system to adapt games based on the user’s performance and abilities. The system also provides users with feedback on their performance, allowing them to track their progress and motivating them to regular training. The entire system is online and is available through a web browser or mobile application. Users can train their cognitive abilities anytime and anywhere, allowing for easy integration into their daily lives. This article [20] addresses the development of a transferable deep learning model for predicting stroke patients’ recovery in various rehabilitation training scenarios. The authors utilize deep learning methods to create a model capable of forecasting the likelihood of patients’ recovery based on specific training regimens. The proposed model demonstrates the ability to transfer its predictive capabilities to different types of rehabilitation programs. The publication [21] deals with the creation of a framework for AI-driven neurorehabilitation training. The author focuses in particular on how to effectively evaluate a patient’s abilities and adapt neurorehabilitation to their needs and abilities. This diagnosis is performed using machine learning methods and algorithms for data analysis. This framework could be used in the future to develop new technologies and applications in the field of neurorehabilitation.

#### Summary

A systematic review of current technologies in neurorehabilitation after traumatic brain injury is provided, for example, in the publication [22]. It is evident that further research is needed to better understand not only the effectiveness of these tools but also the optimal ways to use them for different types of neurological disorders.

It is clear from the above-mentioned survey that published works in the area of neurorehabilitation using ICT techniques (especially artificial intelligence methods) are not quite common, which means that this article will be a quality benefit for future research in this interdisciplinary area.

Four partner entities, specifically the University of Ostrava, Technical University of Ostrava, Ambulance Clinical Psychology, and University Hospital of Ostrava, are collaborating on the development of a unique neurorehabilitation system (called Eddie) for patients with brain damage in the early stages of treatment. The proposed system consists of a hardware interface that interacts with an information system that collects patient data and allows for adaptive adjustment of the entire neurorehabilitation process through uniquely designed algorithms. The authors have chosen two approaches from the field of artificial intelligence: expert systems and artificial neural networks. In this article, we present both approaches and experimentally compare their outputs. Finally, we evaluate their benefits for clinical practice. According to the previous overview, it is not possible to find a corresponding comparable solution for conversions between different diagnostic methods utilizing AI in a similar manner as presented in this article.

### Used ACE-R and CHC

ACE-R (Addenbrooke’s Cognitive Examination-Revised) is a clinical test used to assess cognitive function in patients with suspected memory and cognitive impairment [23]. Since its publication in 2000, ACE-R has become a widely recognized tool for assessing cognitive function. The test consists of five sections that assess different aspects of cognitive function, including attention and orientation, memory, verbal fluency, language abilities, and visuospatial function. Each section has different subtasks and tests that assess different aspects of cognitive function. ACE-R provides scores for each section and an overall score reflecting the patient’s overall cognitive performance. ACE-R is a well-validated and reliable tool for assessing cognitive function, and it is sensitive to detecting cognitive decline associated with neurodegenerative diseases. ACE-R is one of the tools for clinical assessment of patients with suspected cognitive impairment due to its speed, simplicity, and ease of interpreting results.

The Cattell-Horn-Carroll theory (C-H-C) is one of the most significant and complex models of intelligence [24]. This theory divides cognitive abilities into hierarchical layers based on their generality. The first, lowest layer consists of approximately seventy narrow abilities, such as writing and reading speed, verbal fluency, associative memory, articulation speed, and more. These narrow abilities are grouped into more general abilities in the second layer, such as crystallized intelligence (GC), fluid intelligence (GF), quantitative reasoning (GQ), reading and writing ability (GRW), short-term memory (GSM), long-term storage (GLR) and retrieval, visual processing (GV), auditory processing (GA), processing speed (GS), and decision/reaction time/speed (GT). At the highest level of the hierarchy is the general intelligence factor, or g factor, which represents an individual’s overall level of cognitive abilities or intellectual potential. The CHC theory provides a detailed framework for measuring and assessing intelligence and cognitive abilities, but it is a complex model that is often abstract and may be difficult to apply to specific situations.

ACE-R and C-H-C are both models that measure cognitive abilities. While the ACE-R test provides results for five cognitive domains and focuses on cognitive functions associated with dementia and neurological disorders, the C-H-C theory provides a hierarchical model of cognitive abilities that includes multiple layers. While there is some degree of overlap between ACE-R and C-H-C theory, there is no methodology for precisely calculating the results of one test on the results of the other test. The authors of this article proposed such a methodology. The proposed transfer of the CHC domains according to individual outputs of the ACE-R test is shown in Table 1. Outputs from the ACE-R test for each of the five cognitive domains are analysed and translated into CHC abilities. This is described in detail in [3].

**Table 1:** Transfer of the CHC domains according to the ACE-R factor analysis

Transfer ACE-R to C-H-C	GC	GF	GRW	GQ	GSM	GLR	GV	GS	GA
Attention & Orientation	28%	28%		28%	16%				
Memory	15%				39%	46%			
Fluency	25%					25%		50%	
Language	61%		8%						31%
Visuospatial		12%	12%	13%			63%		

### Backpropagation neural network

An artificial neural network is a mathematical approximation of a biological neural network. Its goal is to mimic the function of biological neural networks. Artificial neural networks represent a different approach to problem-solving than conventional computing techniques. A neural network consists of interconnected units, or neurons. The outputs of neurons are fed into the inputs of other neurons. Each neuron transforms its inputs into an output. During the transformation, the neuron can utilize its local memory. Although individual neurons can only perform very simple computations, their strength lies in their collaboration.

The backpropagation neural network is a feed-forward network with one or two hidden layers of neurons. This feed-forward model is called a multilayer perceptron (MLP). In MLP architecture, all neurons from the input layer are connected to all neurons from the first hidden layer. This interconnection pattern remains throughout all layers. The last layer is the output layer, which represents the output from the neural network. Forward propagation in MLP is the following: First, the value of the input neurons is set to the value of the input pattern. Then the states of neurons in hidden layers are computed sequentially. Finally, the output of the network is computed, which is obtained from the output neurons.

In machine learning, backpropagation is a widely used algorithm for training feed-forward neural networks, in which the error propagates from the output back to the input layer during the network adaptation. In order to find the weights (*w*) and biases (*b*) to the desired outputs, the training process is performed on the training data. The training data is passed to the input of the MLP. The actual output is compared with the desired output. Based on the output comparison, the backpropagation algorithm then adjusts the network parameters to better match the desired output. The improvement can be quantified using loss function (1):

1 $$L\left(w,b\right)=\frac1{2n}{\textstyle\sum_x}{\Arrowvert y\left(x\right)-a\Arrowvert}^2$$

where 𝑤 denotes all weights in the MLP, 𝑏 denotes all biases, 𝑛 is the total number of patterns in the training set, 𝑎 is a vector denoting all outputs of the network, 𝑥 denotes the input data. The output 𝑎 depends on the triple (𝑥, 𝑤, 𝑏). 𝑦(𝑥) corresponds to the correct output for a specific input 𝑥. 𝐿 is quadratic loss function, which is represented by mean squared error (MSE). The loss 𝐿(𝑤, 𝑏) decreases. 𝐿(𝑤, 𝑏) ≈ 0 when 𝑦(𝑥) approaches the desired value 𝑎, for all the trained data 𝑥, which is the desired training result. The goal of the backpropagation algorithm is to calculate the partial derivatives $\frac{\partial L}{\partial w}$ and $\frac{\partial L}{\partial b}$ loss function 𝐿 with respect to weights 𝑤 and biases 𝑏. The algorithm presents how much a change in weights or biases in the neural network will change the output of the loss function. The backpropagation method is described in detail, e.g. [25].

### Proposed artificial intelligence models

#### Experimental background

**Fig. 1:**
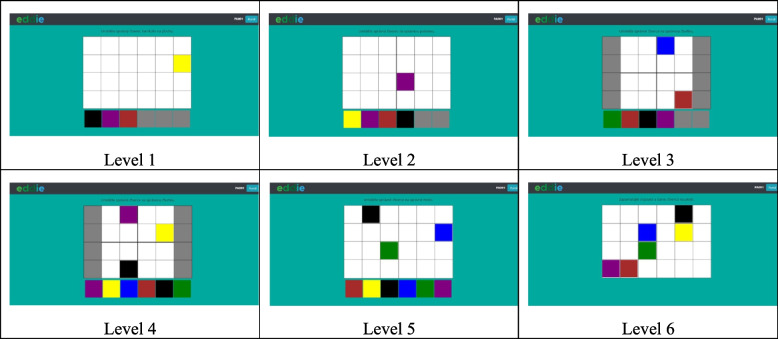
Levels of a long-term memory game

To facilitate the rehabilitation process, the authors of this paper designed and implemented a set of training games for each domain of the CHC model that practice the cognitive skill. Each domain contains six levels of designed games. The patient progresses from the simplest level (level 1) of the game to the most complex (level 6). Example of *training game of long-term memory* is shown in Fig. 1 [26]. The objective is to select correct colourful squares and place them on the right place of the raster.

The results of the ACE-R helped identify the specific CHC domains that needed to be addressed and the ap-propriate level of game to begin rehabilitation in each domain for each patient. The process of assigning appropriate levels of recommended training games to individual patients is very laborious, so the authors of this paper automated it using artificial intelligence tools.

In the following, we detail the use of artificial intelligence tools to initially assign patients to each level of the recommended training games. We describe in detail the proposed models, where a fuzzy expert system and a neural network form the core.

#### Proposed fuzzy expert model

We have developed an intelligent system for recommending rehabilitation procedures for patients, which operates on a foundation of IF-THEN linguistic rules. It is directly implemented in IS Eddie. This system is primarily implemented through LFLC2000, a tool for fuzzy logic rule bases developed at the Institute for Research and Applications of Fuzzy Modeling, University of Ostrava [27].

Fuzzy logic’s strength lies in its capacity to model portions of natural language semantics, making it versatile across various fields such as industry, automation, and expert systems. It utilizes evaluative language expressions and conditional statements embedded with these expressions. Examples of evaluative expressions include “very small”, “more or less medium”, and “roughly large”.

In fuzzy logic, conditional statements containing these evaluative expressions are called fuzzy IF-THEN rules. An example of such a rule is [28]:

$$\mathrm{IF}\;obstacle\;is\;near\;\mathrm{AND}\;car\;speed\;is\;big\;\mathrm{THEN}\;break\;very\;much$$

Such a rule can be schematically written as.

$$\mathrm{IF}\;X_{\mathit1}\;\mathrm{is}\;A_{\mathit1}\;\dots\;\mathrm{AND}\;X_n\;\mathrm{is}\;A_n\;\mathrm{THEN}\;Y\mathit\;is\mathit\;B$$

In our research, we employ rule bases that include antecedent variables derived from five patient test areas. The consequent variable signifies a normalized performance value of a patient across nine standard categories like GC, GF, etc. Each category value helps establish the most appropriate rehabilitation game profile for the patient.

These linguistic descriptions are then used in an approximate reasoning framework. The observations (*X*_*1*_ is *A*_*1*_ AND … AND *X*_*n*_ is *A*_*n*_) generally involve modifications of the antecedent *A*_*i*_ of the rules. These rules, consisting of natural language evaluative expressions, form the foundation for the theory of evaluative linguistic expressions.

We also employ a unique reasoning method and a technique for learning linguistic descriptions from data [29]. Perception-based logical deduction is used as the core inference mechanism, and the Defuzzification of Evaluative Expressions (DEE) method [27] for defuzzification.

Evaluative linguistic expressions used within fuzzy IF-THEN rules follow this structure: [(sign)](linguistic modifier)(basic expression). Basic expressions include “small”, “medium”, and “big”, while linguistic modifiers range from “extremely” to “very very roughly”.

When a rule is learned from a single observation, the best fitting linguistic term for a specific value is sought. For example, within the GC category and from data observations, particular rules are obtained. These raw learned rules are subsequently filtered to remove redundancies and inconsistencies. Any duplicate rules are automatically excluded during the linguistic learning process.

**Fig. 2:**
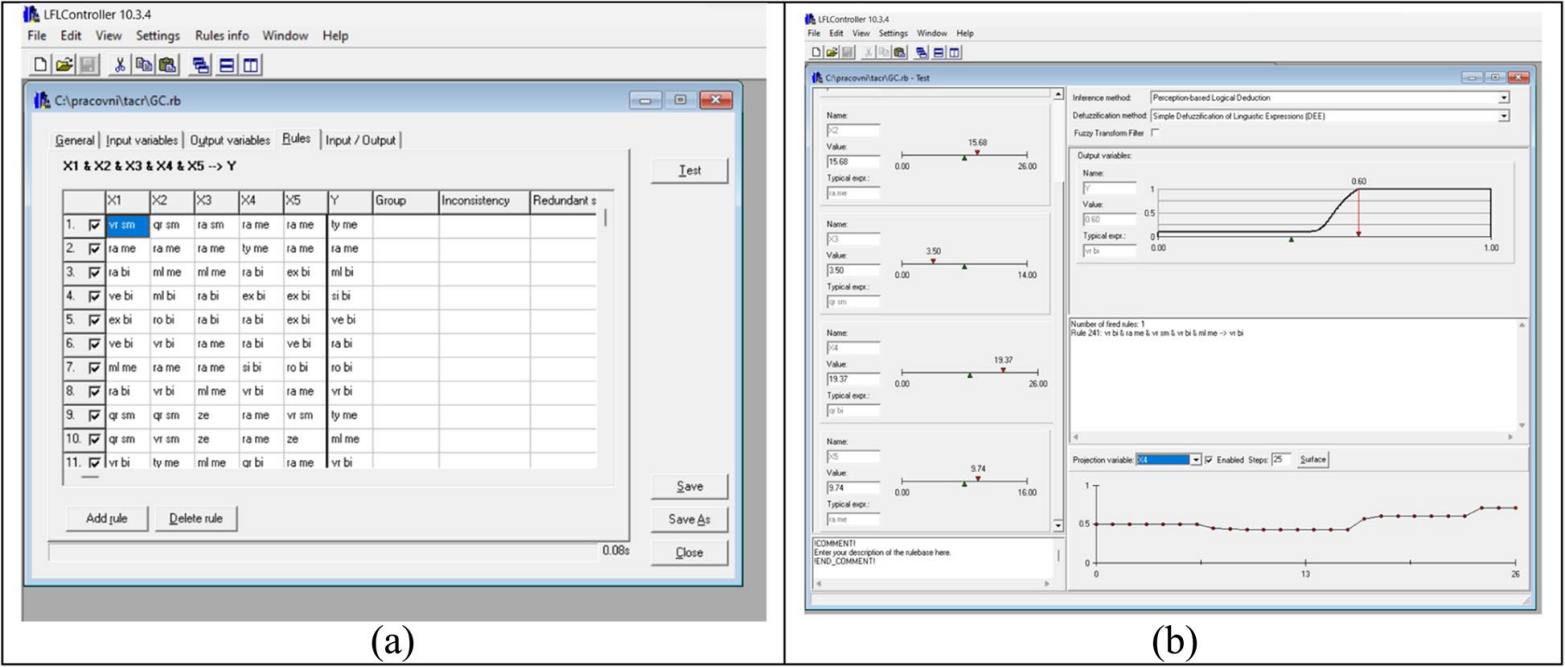
**a** LFLC GUI interface of GC learned rulebase with particular rules (5 input and output GC score). **b** LFLC GUI interface of GC learned rulebase testing environment (fired rule and variable projection)

We used the default settings of evaluative expressions, which permit a limited use of modifiers with atomic expressions. While it’s possible to alter the parameters of modifiers, we opted to stick with the default settings in our work, as detailed in the article concerning the proposed expert system [3] Fig. 2.

#### Proposed neural networks model

**Fig. 3:**
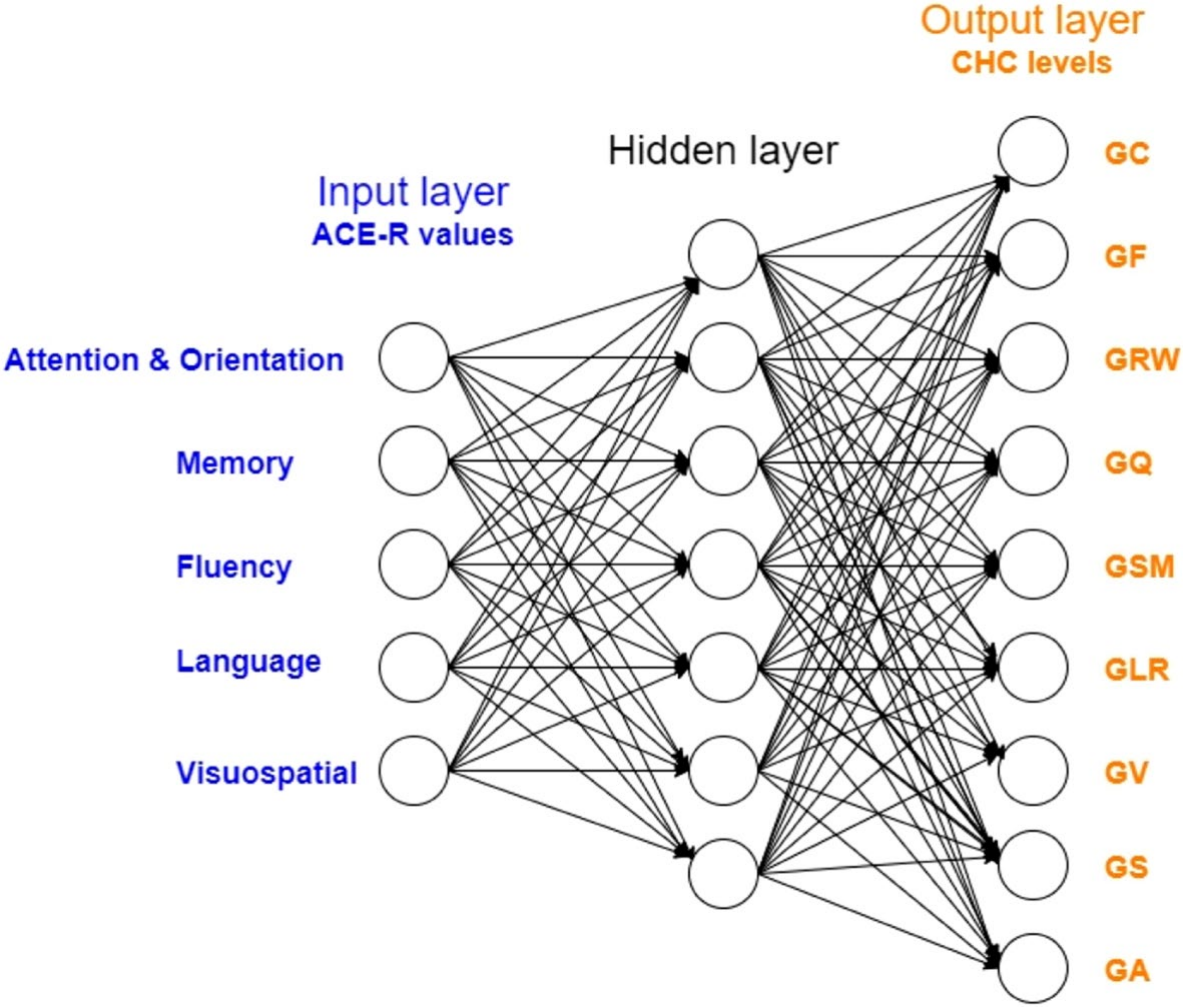
The used neural network topology 5-7-9. There are 5 neurons in the input layer, where each represents a value of the ACE-R test result. There are 9 neurons in the output layer, where each neuron represents a specific area of the CHC model of intelligence: crystallized intelligence (GC), fluid intelligence (GF), reading and writing ability (GRW), quantitative reasoning (GQ), short-term memory (GSM), long-term storage (GLR) and retrieval, visual processing (GV), processing speed (GS), and auditory processing (GA)

We have chosen a multilayer neural network with topology of 5-7-9 (Fig. 3). This means that there are 5 neurons in the input layer, where each represents a value of the ACE-R test result that has been normalized to an interval 〈0,1〉. In the output layer, we have 9 neurons, where each neuron represents a specific area of the CHC model of intelligence. Each value is divided into 6 segments (according to the levels of the game) and all values are normalized to the interval 〈0,1〉. The input values were adapted to the needs of adaptation using the backpropagation method with a sigmoid activation function. To estimate the number of neurons in the hidden layer, we used [30] ${N}_{h}=\sqrt{{N}_{i}\cdot {N}_{o}}$, i.e. the number of hidden neurons *N*_*h*_ should be between the size of the input layer (*N*_*i*_ – number of input neurons) and the size of the output layer (*N*_*o*_ – number of output neurons).

The training data consisted of a sample of 1000 uniformly machine-generated input vectors representing the outputs of the ACE-R test. According to the proposed expert calculation (Table 1), we determined the values of the output vectors for each input. The testing data comprised 703 real patient data.

As an activation function, the standard (logistic) sigmoid with a steepness parameter α = 1 was used. The learning rate α was adjusted during adaptation as follows: α = 1 for cycles 1-1000, α = 0.5 for cycles 1001–2000, and α = 0.1 for cycles greater than 2000. These values of the learning rate α were set based on experimental studies. After every 1000 cycles, the program paused adaptation and reduced the learning rate α to a smaller value, resulting in smoother weight updates. As a result, the algorithm initially finds the solution region “roughly,“ and then adaptation becomes more precise, and weight values change more gradually. The termination condition for the adaptation algorithm was a specified threshold value for the network’s total error E ≤ 0.07, which is nearly perfect learning of the entire training set.

The neural network is directly implemented in IS Eddie, so it is not necessary to use third-party software for its evaluation, unlike the proposed fuzzy expert model, where it is necessary to run LFLC. Data evaluation using the neural network is always performed for one patient at a time, enabling very fast calculations. Due to the speed of the neural network evaluation, there is no need to store the controlled data in the database, and the evaluation can take place before each patient rehabilitation selection page is opened without any impact on performance and page loading speed.

### Experiments and algorithms comparison

The cognitive rehabilitation took place at the workplace of the University Hospital of Ostrava. The test group included 703 patients, 351 of whom were men and 352 women. The age range of patients was 19–97 years. Concerning their education, the distribution is the following: 126 patients have primary school, 182 have vocational certificate, 254 have secondary school, and 141 patients have university degree. The both proposed artificial intelligence models was adapted on real data, which we compared against psychologists’ expert knowledge for game level recommendation. We compared the recommended levels of neurorehabilitation games by expert knowledge (psychologists) vs. neural network level prediction vs. fuzzy expert system prediction. The recommendation is based on 5 measured ACE-R scores.

### Performance comparison of FES and NN

The neural network model performed according to expert knowledge for every CHC category except for GSM as shown in Table 2 (level 0 means matching psychologists’ recommended level), where is also higher rate of incorrect prediction for level above psychologists’ prediction. Table 2 shows very good results for CHC factors GC, GF, GQ, GS and GA (above 90% cases matching psychologists’ prediction). Tables 2 and 3 represent level differences:

- Level error < = 0 – cases where the difference between NN predicted game level is same or lower level than expert’s knowledge evaluation.
- Level error > 0 – cases where the difference between NN predicted game level is higher level than expert’s knowledge evaluation (the worse situation since the patient may start at too complex level).

**Table 2:** Levels comparison difference ratio. Comparison Expert Knowledge (psychologists) vs. Neural NLtwork Levels prediction (NN)

NN - experts	GC	GF	GRW	GQ	GSM	GLR	GW	GS	GA
Level error < = 0	694	689	686	689	596	658	676	703	693
Level error > 0	9	14	17	14	107	45	27	0	10
Total observations	703	703	703	703	703	703	703	703	703
Level error > 0	**1.28%**	**1.99%**	**2.42%**	**1.99%**	**15.22%**	**6.40%**	**3.84%**	**0.00%**	**1.42%**

The Fuzzy Expert System model performed according to expert knowledge for every CHC category except for GSM and GLR as shown in Table 3, where is also higher rate of incorrect prediction for level above psychologists’ prediction. Table 3 shows very good results for CHC factors GC, GRW, and GA (above 90% cases matching psychologists’ prediction).

**Table 3:** Levels comparison difference ratio. Comparison Expert Knowledge (psychologists) vs. Fuzzy Expert System Levels prediction (FES)

FES - experts	GC	GF	GRW	GQ	GSM	GLR	GW	GS	GA
Level error < = 0	689	676	697	674	622	621	674	603	694
Level error > 0	14	27	6	29	81	82	29	100	9
Total observations	703	703	703	703	703	703	703	703	703
Level error > 0	**1.99%**	**3.84%**	**0.85%**	**4.13%**	**11.52%**	**11.66%**	**4.13%**	**14.22%**	**1.28%**

For proper rehabilitation process it is more important not to predict higher levels without appropriate scores. It means our main result was to predict with zero differences or negative differences. The performed experiments give similar results also according to this criterion (difference 0 or below). Tables 2 and 3 provide relevant explanation considering only levels difference below and including 0 (important for correct neurorehabilitation process) and levels difference above 0 (incorrectly assigned too high level). If the patient is assigned level above his real CHC factor performance, it can slow down curing process (in this case he can start at level beyond his capabilities).

### Evaluation of neural network outputs in detail

Statistical validation for recommendation levels results shown the only statistically significant difference between NN and psychologists’ recommendation for GSM based on ANOVA and Kruskal-Wallis (K-W) test. We have performed analysis of game level recommendation for every particular CHC factor against psychologists’ recommended values.

**Fig. 4:**
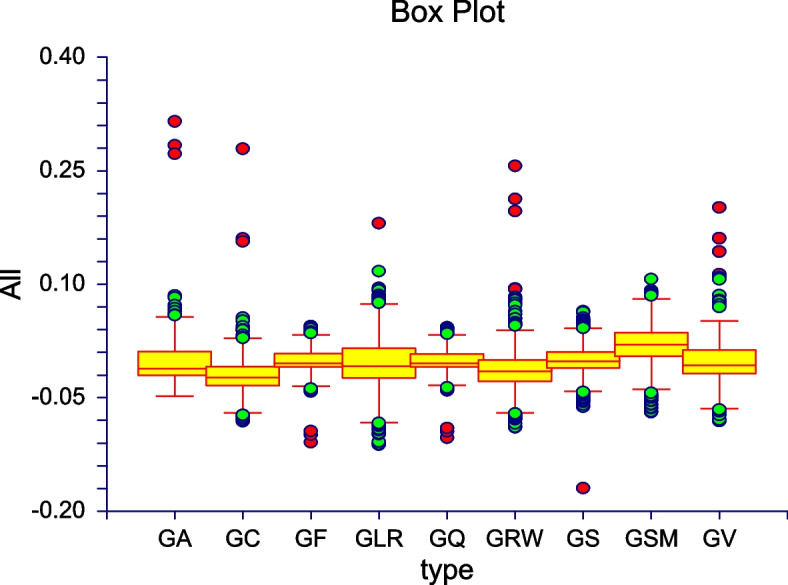
Evaluation of individual CHC modules by NN showing differences of NN and expert’s evaluation in Box-plot

Table 4 demonstrates that for all CHC factors the difference is not statistically significant except for GSM, where K-W test allowed us to reject the zero hypothesis on 5% level (even the results is borderline and standard ANOVA allows to accept zero hypothesis). It corresponds with previous table data, since prediction for GSM had several times higher rate of false prediction than for average of CHC factors. If we compare the results of NN recommendation difference of predicted performance of subject in particular factor of CHC, we also observe statistical differences between CHC factors (difference of 0–1 result of psychologists’ calculation and NN prediction of performance 0–1 in CHC factor). Again, we can see anomalous behaviour for GSM factor. Obviously both ANOVA (Table 5) and K-W test enables us to reject zero hypothesis about same results for all CHC factors (Fig. 4).

**Table 4:** Statistical significance of difference between expert knowledge (psychologists’) and neural network levels prediction

Levels	GC	GF	GRW	GQ	GSM	GLR	GW	GS	GA
Expert mean	5.14082	5.17212	5.36273	5.16643	4.20910	3.84921	5.06116	3.66856	5.60455
NN mean	5.03556	5.16358	5.29587	5.16500	4.35988	3.85490	5.06543	3.62019	5.60597
Difference	0.10526	0.00853	0.06685	0.00142	-0.15078	-0.00569	-0.00427	0.04836	-0.00142
*P*-value (ANOVA)	0.06262	0.88297	0.21427	0.98041	0.04458	0.94340	0.95064	0.59210	0.97476
*P*-value (K-W)	0.06131	0.77292	0.44191	0.88069	0.05748	0.92845	0.56774	0.55772	0.82942
Stat.signif. 5% (ANOVA)	No	No	No	No	Yes	No	No	No	No
Stat.signif. 5% (K-W)	No	No	No	No	No	No	No	No	No
	ACCEPT	ACCEPT	ACCEPT	ACCEPT	REJECT	ACCEPT	ACCEPT	ACCEPT	ACCEPT

**Table 5:** ANOVA results for difference of CHC factors

Expected Mean Squares Section						
Source		Term	Denominator	Expected		
Term	DF	Fixed?	Term	Mean Square		
A: type	8	Yes	S(A)	S + sA		
S(A)	6318	No		S(A)		
Expected Mean Squares are for the balanced cell-frequency case.						
Analysis of Variance Table						
Term	DF	Sum of Squares	Mean Square	F-Ratio	Prob Level	Power (α = 0.05)
A: type	8	0.607587	7.59E-02	115.12	0.000000*	1
S(A)	6318	4.16824	6.60E-04			
Total (Adjusted)	6326	4.775826				
Total	6327					

*< 0.0000001

Bonferroni comparison test (Table 6) showed mainly GSM factor results differences against all other factors of CHC and also differences between GC against all other and GRW against all other factors.

**Table 6:** Bonferroni (All-Pairwise) Multiple Comparison test results for difference of CHC factors

Bonferroni (All-Pairwise) Multiple Comparison Test			
Response: All			
Term A: type			
Alpha = 0.050 Error Term = S(A) DF = 6318 MSE = 6.597404E-04 Critical Value = 3.1970			
			Different From
Group	Count	Mean	Groups
GC	703	-2.030576E-02	GRW, GA, GLR, GV, GF, GQ, GS, GSM
GRW	703	-0.0131983	GC, GA, GLR, GV, GF, GQ, GS, GSM
GA	703	-4.191941E-03	GC, GRW, GSM
GLR	703	-3.579397E-03	GC, GRW, GSM
GV	703	-2.59945E-03	GC, GRW, GSM
GF	703	-1.274888E-03	GC, GRW, GSM
GQ	703	-1.272578E-03	GC, GRW, GSM
GS	703	-1.905147E-04	GC, GRW, GSM
GSM	703	1.810134E-02	GC, GRW, GA, GLR, GV, GF, GQ, GS

### Analysis of the experimental part

**Fig. 5:**
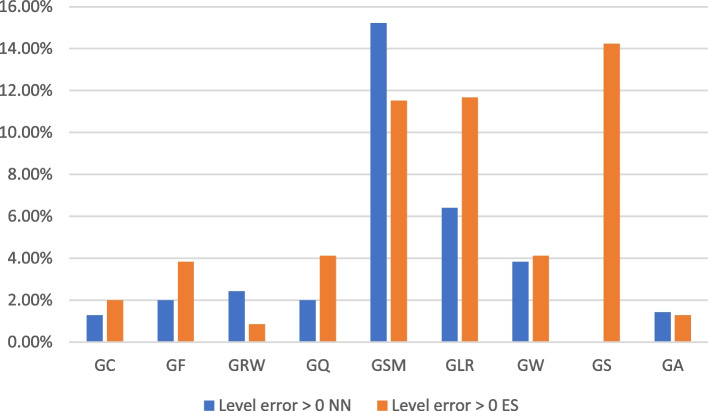
Performance comparison of ES and NN

If we compare results of FES in Table 3 with results of NN for game levels prediction (Table 2), we can observe overall better results for NN, with some exceptions – GRW and GSM (Fig. 5). But even better FES result for GSM is of still similarly high failure rate. It should also be noted that for GS factor NN provides 100% results and FES worst result for all CHCs (only about 84% success rate).

**Table 7:** Statistical significance of difference between expert knowledge (psychologists’) and fuzzy expert systems levels prediction

Levels	GC	GF	GRW	GQ	GSM	GLR	GW	GS	GA
Expert mean	5.1408	5.1721	5.3627	5.1664	4.2091	3.8492	5.0612	3.6686	5.6046
FES mean	4.9218	5.0071	5.1522	5.0057	4.0996	3.8606	4.8606	3.6700	5.3954
Difference	0.2191	0.1650	0.2105	0.1607	0.1095	-0.0114	0.2006	-0.0014	0.2091
*P*-value (ANOVA)	0.0002	0.0055	0.0001	0.0069	0.1332	0.8829	0.0031	0.9873	0.0000
*P*-value (K-W)	0.0005	0.0044	0.0002	0.0062	0.0575	0.9961	0.0002	0.7626	0.0000
Stat.signif. 5% (ANOVA)	Yes	Yes	Yes	Yes	No	No	Yes	No	Yes
Stat.signif. 5% (K-W)	Yes	Yes	Yes	Yes	No	No	Yes	No	Yes
	REJECT	REJECT	REJECT	REJECT	ACCEPT	ACCEPT	REJECT	ACCEPT	REJECT

Table 7 shows statistical results for FES in similar way like for NN. Note that for neurorehabilitation process, the level error > 0 parameter is more important for the recommendation of particular case, than overall statistical significance. We present these data analysis results to stress differences between FES and NN methods. Statistically significant differences for NN model against experts’ knowledge (not considering only positive error level) are only for GSM CHC type. On the other hand, FES produced statistically significant differences for GC, GF, GRW, GQ, GW and GA. We can observe statistical better performance of FES only for GSM type in contrast to NN.

**Table 8:** Advantages and disadvantages of FES and NN response according to CHC type (pure statistical analysis and level error analysis)

CHC type	FES difference overall	NN difference overall	Level error > 0 adv.
GC	REJECT	ACCEPT	NN+
GF	REJECT	ACCEPT	NN+
GRW	REJECT	ACCEPT	ES+
GQ	REJECT	ACCEPT	NN+
GSM	ACCEPT	REJECT (K-W)	ES+
GLR	ACCEPT	ACCEPT	NN+
GW	REJECT	ACCEPT	similar
GS	ACCEPT	ACCEPT	NN++ (no error)
GA	REJECT	ACCEPT	similar

Table 8 shows better results of NN model in more CHC types not only in terms of statistical significance of pure differences of levels, but also in most of CHC types. If both the parameters are taken into account, NN model has significant disadvantage only in GSM type. We consider that the significant increase in the error rate in GSM could be caused by an inaccurately designed expert coefficient for the transfer of the CHC domains according to individual outputs of the ACE-R test (see Table 1) or an error in the process of diagnosing a real patient in a given domain. In any case, it is an interesting and important finding that our proposed transfer model identified a potential inaccuracy or incorrectness in the real patient diagnosis process. Refinement of the proposed conversion model will be the subject of our future work. Table 9 provides also differences in level evaluation between FES and NN.

**Table 9:** Statistical significance of difference between fuzzy expert systems and neural network levels prediction

Levels	GC	GF	GRW	GQ	GSM	GLR	GW	GS	GA
FES mean	4.9218	5.0071	5.1522	5.0057	4.0996	3.8606	4.8606	3.6700	5.3954
NN mean	5.0356	5.1636	5.2959	5.1650	4.3599	3.8549	5.0654	3.6202	5.6060
Difference	-0.1138	-0.1565	-0.1437	-0.1593	-0.2603	0.0057	-0.2048	0.0498	-0.2105
*P*-value (ANOVA)	0.0525	0.0082	0.0112	0.0071	0.0003	0.9418	0.0029	0.5764	0.0000
*P*-value (K-W)	0.0979	0.0099	0.0045	0.0093	0.0002	0.9434	0.0000	0.7498	0.0000
Stat.signif. 5% (ANOVA)	No	Yes	Yes	Yes	Yes	No	Yes	No	Yes
Stat.signif. 5% (K-W)	No	Yes	Yes	Yes	Yes	No	Yes	No	Yes
	ACCEPT	REJECT	REJECT	REJECT	REJECT	ACCEPT	REJECT	ACCEPT	REJECT

## Conclusion and future work

This article presents a proposed intelligent neurorehabilitation system called Eddie, which contributes to the systematization and increased effectiveness of care for patients with acquired brain injury from acute treatment phases through the first six months. The core of this system is formed by artificial intelligence algorithms: fuzzy expert systems and artificial neural networks. The neurorehabilitation system Eddie is the output of a research project financially supported by the Technology Agency of the Czech Republic (TAČR). The entire proposed neurorehabilitation system is available at the following link: (https://eddie.osu.cz).

In the process of diagnosis and rehabilitation, it is extremely important to initiate targeted patient treatment as soon as possible and in the best possible way. Available certified diagnostic methods are often impossible to deploy in acute conditions for a variety of reasons. It is therefore of great benefit to be able to use one diagnostic technique with subsequent validated conversion to other diagnostic systems. For this conversion we can utilize expert-defined conversion relations. However, we are no longer able to verify the quality and relevance of these relations. Therefore, it is advisable to subject the expert-defined conversion relations to an external validation tool, which in our case, is represented by the neural network we have defined. Since neural networks are capable of generalization, we use this capability to detect and quantify any imperfections and inaccuracies that the expert may commit when designing the transfer relations.

In the article [3], we describe in detail the use of fuzzy expert systems for initial patient classification into recommended training game levels. This article builds upon that work and aims to describe and experimentally verify the proposed Eddie neurorehabilitation system, whose core is artificial neural networks. Both proposed models were tested on the same dataset containing 703 samples, and their experimental outputs were compared and statistically evaluated with respect to the conclusions of experts that were available. From the graphs and tables presented in Chap. 6, it is evident that the smart neurorehabilitation system using artificial neural networks achieved significantly better results than the neurorehabilitation system whose core is a fuzzy expert system. The outputs of the proposed neurorehabilitation system thus allow for the automatic creation of a personalized and optimized neurorehabilitation plan for individual patients. For each patient, the results of ACE-R helped identify specific CHC domains that need to be rehabilitated, as well as the initial game level for rehabilitation in each domain.

Interestingly and importantly, our proposed conversion model (Table 1) identified a potential inaccuracy or incorrectness in the process of diagnosing real patients. Refinement of the proposed inter-model conversion will be the subject of our future work. According to related works (Chap. 2), it is not possible to find a corresponding comparable solution for conversions between different diagnostic methods utilizing AI in a similar manner as presented in this article.

In particular, our next work will primarily focus on confirming and improving an apparatus of patient diagnosis in cooperation of individual experts in the field with a classification model based on methods not only from neural networks but prospectively from other AI domains or statistical models.

In future work, the authors of this article also will focus on deploying the proposed smart neurorehabilitation system Eddie into full operation at the neurological clinic in University Hospital Ostrava and evaluating its effectiveness on obtained patient data.

## Data Availability

Not applicable.

## Abbreviations

ACE- R: Addenbrooke's cognitive examination
CHC: Cattell – Horn – Carroll intelligence model
CNN: Convolutional Neural Network
ES: Expert System
GC: Crystallised Intelligence
GF: Fluid Intelligence
GQ: Quantitative Reasoning
GRW: Reading and Writing Ability
GSM: Short-Term Memory
GLR: Long Term Storage and Retrieval
GV: Visual Processing
GA: Auditory Processing
GS: Processing Speed
GT: Decision/Reaction Time/Speed
K-W: Kruskal-Wallis
LFLC2000: Linguistic Fuzzy Logic Controller
LSTM: Long short-term memory neural network
MEC: Mobile Edge Computing
MMSE: Mini Mental State Exam
NR: Neurocognitive rehabilitation
RBM: Restricted Boltzmann Machine
TCN: Temporal Convolution Network

## References

[1] Wilson BA (2003). Goal planning rather than neuropsychological tests should be used to structure and evaluate cognitive rehabilitation. Brain Impair.

[2] Connolly ML, Bowden SC, Simpson LC, Horne M, McGregor S (2020). The latent-variable structure of the Addenbrooke’s cognitive examination-revised. Arch Clin Neuropsychol.

[3] Kotyrba M, Habiballa H, Volná E, Jarusek R, Smolka P, Prasek M, Malina M, Jaremova V, Vantuch J, Bar M, Kulistak P (2023). Expert System for Neurocognitive Rehabilitation based on the transfer of the ACE-R to CHC Model factors. Mathematics.

[4] Laver KE, Lange B, George S, Deutsch JE, Saposnik G, Crotty M. Virtual reality for stroke rehabilitation. Cochrane Database of Systematic Reviews. 2017;11. 10.1002/14651858.CD008349.pub4. 10.1002/14651858.CD008349.pub4 PMC6485957 29156493

[5] Aulisio MC, Han DY, Glueck AC (2020). Virtual reality gaming as a neurorehabilitation tool for brain injuries in adults: a systematic review. Brain Injury.

[6] Srivastav AK, Samuel AJ (2020). E-Neurorehabilitation: use of mobile phone based health applications during the COVID-19 pandemic. J Rehabil Med.

[7] Chen J, Jin W, Zhang XX, Xu W, Liu XN, Ren CC (2015). Telerehabilitation approaches for stroke patients: systematic review and meta-analysis of randomized controlled trials. J Stroke Cerebrovasc Dis.

[8] Messinis L, Kosmidis MH, Nasios G, Dardiotis E, Tsaousides T (2019). Cognitive neurorehabilitation in acquired neurological brain injury. Behav Neurol.

[9] Díez-Cirarda M, Ibarretxe-Bilbao N, Peña J, Ojeda N (2018). Neurorehabilitation in Parkinson’s disease: a critical review of cognitive rehabilitation effects on cognition and brain. Neural Plast.

[10] Rizzo A, Bouchard S (2019). Virtual reality for psychological and neurocognitive interventions.

[11] Moreno A, Wall KJ, Thangavelu K, Craven L, Ward E, Dissanayaka NN (2019). A systematic review of the use of virtual reality and its effects on cognition in individuals with neurocognitive disorders.

[12] Klimova B (2016). Computer-based cognitive training in aging. Front Aging Neurosci.

[13] Yang S, Li R, Li H, Xu K, Shi Y, Wang Q, Yang T, Sun X (2021). Exploring the use of brain-computer interfaces in stroke neurorehabilitation. Biomed Res Int.

[14] Templeton JM, Poellabauer C, Schneider S (2020). Enhancement of neurocognitive assessments using smartphone capabilities: Systematic review. JMIR mHealth and uHealth.

[15] Fazekas G, Tavaszi I (2019). The future role of robots in neuro-rehabilitation. Expert Rev Neurother.

[16] Channa A, Popescu N, Ciobanu V (2020). Wearable solutions for patients with Parkinson’s disease and neurocognitive disorder: a systematic review. Sensors.

[17] Solana J, Caceres C, Garcia-Molina A, Opisso E, Roig T, Tormos JM, Gomez EJ (2014). Improving brain injury cognitive rehabilitation by personalized telerehabilitation services: Guttmann neuropersonal trainer. IEEE J Biomed Health Inf.

[18] Jung HT, Daneault JF, Lee H, Kim K, Kim B, Park S, Ryu T, Kim Y, Lee SI (2019). Remote assessment of cognitive impairment level based on serious mobile game performance: an initial proof of concept. IEEE J Biomedical Health Inf.

[19] Walton CC, Lampit A, Boulamatsis C, Hallock H, Barr P, Ginige JA, Valenzuela M (2019). Design and development of the brain training system for the digital maintain your brain dementia prevention trial. JMIR Aging.

[20] Lin, P. J., Zhai, X., Li, W., Li, T., Cheng, D., Li, C., … Ji, L. (2022). A Transferable Deep Learning Prognosis Model for Predicting Stroke Patients’ Recovery in Different Rehabilitation Trainings. IEEE Journal of Biomedical and Health Informatics, 26(12),6003–6011. 10.1109/JBHI.2022.3205436 36083954

[21] Rodrigues PAG. (2022) A framework for AI-driven neurorehabilitation training: the profiling challenge, Doctoral dissertation, Universidade da Madeira.

[22] Bonanno M, De Luca R, De Nunzio AM, Quartarone A, Calabro RS (2022). Innovative technologies in the neurorehabilitation of traumatic brain injury: a systematic review. Brain Sci.

[23] Mathuranath PS, Nestor PJ, Berrios GE, Rakowicz W, Hodges JR (2000). A brief cognitive test battery to differentiate Alzheimer’s disease and frontotemporal dementia. Neurology.

[24] McGrew K (2009). CHC theory and the human cognitive abilities project: standing on the shoulders of the giants of psychometric intelligence research. Intelligence.

[25] Hecht-Nielsen R (1992). Theory of the backpropagation neural network. Neural networks for perception.

[26] Martinkova L, Prasek M, Kotyrba M, Volna E. (2022, April). Application for training long-term memory on the basis of the CHC intelligence model. In *AIP Conference Proceedings* (Vol. 2425, No. 1). AIP Publishing.

[27] Dvorak A, Habiballa H, Novak V, Pavliska V (2003). The software package LFLC 2000-its specificity, recent and perspective applications. Comput Ind.

[28] Novak V (2007). Mathematical fuzzy logic in modeling of natural language semantics. Fuzzy logic.

[29] Belohlavek R, Novak V (2002). Learning rule base in linguistic expert systems. Soft Comput.

[30] Vujičić T, Matijevi T, Ljucović J, Balota A, Ševarac Z. (2016). Comparative analysis of methods for determining number of hidden neurons in artificial neural network. In *Central European conference on information and intelligent systems* (Vol. 219), 2019 – 223.

